# Filiform papillae do not have taste buds: reply

**DOI:** 10.1590/S1808-86942012000400003

**Published:** 2015-10-20

**Authors:** Marilda Aparecida Milanez Morgado de Abreu, Luc Louis Maurice Weckx, Cleonice Hitomi Watashi Hirata

**Affiliations:** M.Sc; Graduate student; Full Professor - Department of Otorhinolaryngology and Head and Neck Surgery; PhD. Head of the Stomatology Sector. Federal University of São Paulo

**Keywords:** histology, taste bud, tongue

**Figure 3:**
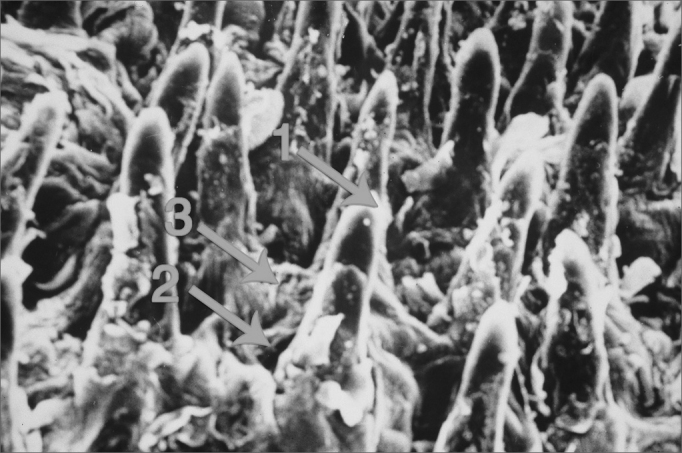
Filiform papillae and fungiform papillae with taste bud on top (1: filiform papilla; 2: fungiform papilla; 3: Taste bud).

We thank you for your comments published in the Brazilian Journal of Otorhinolaryngology: “Histological and ultra-structural aspects of undernourished rats' tongues. Braz J Otorhinolaryngol. vol. 72 $n^{\underline{o}}$ 4, São Paulo, July/August 2006”. It is true that filiform papillae do not have taste buds. However, since the paper was originally published in Portuguese, there was a misinterpretation of what we wanted to say when it was translated into English. This happened because we were not very clear on the way we described the legend in Figure 3. We meant to say that: “filiform papilla and fungiform papilla with taste bud on top” In other words, the taste bud is associated only to the latter. We added arrows to the figure in order to identify the structures.

Sincerely,

*Marilda Aparecida Milanez Morgado de Abreu Luc Louis Maurice Weckx Cleonice Hitomi Watashi Hirata*

